# Bibliometric analysis of medicine-related publications on poverty (2005–2015)

**DOI:** 10.1186/s40064-016-3593-3

**Published:** 2016-10-28

**Authors:** Waleed M. Sweileh, Samah W. Al-Jabi, Ansam F. Sawalha, Adham S. AbuTaha, Sa’ed H. Zyoud

**Affiliations:** 1 Department of Physiology, Pharmacology, and Toxicology, College of Medicine and Health Sciences, An-Najah National University, Nablus, Palestine; 2 Department of Clinical and Community Pharmacy, College of Medicine and Health Sciences, An-Najah National University, Nablus, Palestine

**Keywords:** Poverty, Bibliometric analysis, Health journals

## Abstract

**Background:**

Poverty is a global problem. The war against poverty requires not only financial support, but also poverty-related research to pinpoint areas of high need of intervention. In line with international efforts to fight poverty and negative consequences, we carried out this study to give a bibliometric overview of medicine-related literature on poverty. Such a s study is an indicator of the extent of interaction of various international key players on the war against poverty-related health problems.

**Methods:**

Scopus was used to achieve the objective of this study. The time span set for this study was 2005–2015. Poverty-related articles under the subject area “Medicine” were used to give bibliometric indicators such as annual growth of publications, international collaboration, highly cited articles, active countries, institutions, journals, and authors.

**Results:**

The total number of retrieved articles was 1583. The Hirsh-index of retrieved articles was 56. A modest and fluctuating increase was seen over the study period. Visualization map of retrieved articles showed that “HIV”, infectious diseases, mental health, India, and Africa were most commonly encountered terms. No significant dominance of any particular author or journal was observed in retrieved articles. The United States of America had the largest share in the number of published articles. The *World Health Organization* and *Centers for Disease Prevention and Control* were among top active institutions/organizations. International collaboration was observed in less than one third of publications. Top cited articles focused on three poverty-related health issues, mainly, infectious diseases, malnutrition, and child development/psychology. Most of top articles were published in high impact journals.

**Conclusions:**

Data indicated that articles on poverty were published in high influential medical journals indicative of the importance of poverty as a global health problem. However, the number publications and the extent of international collaborations was lower than expected given the huge burden of poverty-related health problems.

## Background

Poverty is a global problem and the fight against poverty is a worldwide responsibility (Ferreira and Ravallion 2008; Kim and Chan 2013). Poverty is present everywhere, even in developed countries (Pritchett 2014). According to World Health Organization (WHO), more than one billion people in the word have an income of less than one dollar a day. The WHO is working with poor countries to implement health policies that prioritize health needs of poor people (World Health Organization 2016). A wide range of communicable and non-communicable diseases such as parasitic, nutritional, neurodevelopmental and cardiovascular diseases have been linked to poverty (Kalichman et al. 2014; Marmot 2016). At the global level, acquired immune deficiency syndrome (AIDS), malaria, and tuberculosis are major poverty-related diseases. These diseases are found mainly in poor developing countries such as sub-Saharan Africa (Buve et al. 2002; MacDonald 2005; Whiteside 2010; von Philipsborn et al. 2015). Many of neglected tropical diseases such as leprosy, lymphatic filariasis, chagas disease, trypanosomiasis, onchocerciasis, schistosomiasis, helminthiasis, leishmaniasis, and trachoma are mainly present in poor communities (Hotez et al. 2006, 2008; Feasey et al. 2010; Hotez et al. 2012). Centers for Disease Control and Prevention (CDC) have identified these diseases as public health priority given the large number of people infected with these diseases (Centers for Disease Prevention and Control 2016). Poor nutrition, respiratory diseases, and cardiovascular diseases are major non-communicable diseases associated with poverty. It is estimated that one third of children in sub-Saharan Africa showed physical signs of malnutrition (Piwoz and Preble 2000). Malnutrition can negatively affect the immune system and thus increase the risk of human immunodeficiency virus infection and its transmission from mothers to newborn (Friis and Michaelsen 1998). Poverty itself can be considered as major risk factor for many diseases and as a barrier for economic development (de la Barra 1998; Piazza 2006; Hilson 2009).

An important aspect in the war against poverty is research that sheds light on poverty and its association with physical and mental health especially in children and pregnant women. In fact, at least four peer reviewed journals are specialized in publishing research related poverty. Such journals include, *Infectious Diseases of Poverty*, *Journal of Poverty*, *The Journal of Poverty and Social Justice*, and *Journal of Children and Poverty.* Publications on health-related aspects of poverty constitute a database for each country in order to formulate its strategic health plans and to prioritize its agenda accordingly. Poverty is considered a top agenda for many international health agencies. Actually, the Millennium Development Goals (MDG) launched in year 2000 by the United Nations had eight goals to be accomplished in year 20015. One of these goals, the first goal, was to eradicate extreme poverty and hunger. The MDG is now over and it is time to assess how countries, institutions, and international agencies reacted to this goal in the past years from a research and publication point of view. The volume of literature produced on poverty-related health issues is considered an indicator of international responsibility toward poor countries and an indicator of the amount of efforts implemented to fight poverty-related diseases. In line with international efforts to eliminate poverty and its related medical and health consequences, we participated in this study to assess research growth and highlight most important topics on poverty-related health issues. Furthermore, this study is part of global efforts needed to shed light on poverty as a public health issue.

Bibliometric analysis is a tool used to assess the quantity and quality of research output on a certain topic (Thompson and Walker 2015). Bibliometric analysis on medicine-related poverty literature will give an idea on the volume of literature on this topic and content of articles that are being mostly cited in this field. Furthermore, bibliometric analysis will shed light on network of authors and co-authorship that will help finding research partners across the globe for potential collaboration and joint grant seeking. Bibliometric analysis is also an important indicator of the impact of governmental and non-governmental initiatives on war against poverty and its consequences (Thompson and Walker 2015; Thompson and Clark 2015). Therefore, the objective of this study was to give a bibliometic overview on medicine-related literature on poverty using Scopus search engine. Specifically, the number of publications, top active countries and institutions, highly cited articles, citation analysis, international collaboration, top active authors, and journals involved in publishing articles on poverty will be presented.

## Methods

This study was carried out using one of the largest and up-to-date electronic databases, Scopus. In bibliometric studies, researchers can use any of the existing databases such as PubMed or Web of Science (WoS) or Pubmed to retrieve required data. It is the authors understanding that Scopus offers advantages over other databases. For example, the volume of literature available through Scopus is higher than that available through PubMed or WoS. Furthermore, Scopus provides citation analysis and a friendly search engine. Scopus is produced by Elsevier and covers more than 20,000 journals and has 100% Medline coverage. Scopus offers about 20% more coverage than WoS, whereas Google Scholar offers results of inconsistent accuracy (Falagas et al. 2008). Discussion regarding these databases is beyond the scope of this manuscript. Further details and comparison of advantages and disadvantages of each database have been published (Falagas et al. 2008).

In this study, poverty was the only concept in this study. However, we did not search for all publications on poverty. Rather, search was limited to medicine-related publications on poverty. To achieve this, search strategy was based on poverty concept followed by limiting the findings to publications in journals categorized in Scopus as “Medicine”. In this search strategy, we do not need to do intersection between results obtained from different concepts. The approach was made easy and reproducible using Scopus functions which can help in limiting and refining results to achieve the required goal. For example, Scopus has a function called “Subject” which includes “medicine” as well as other subjects that the researcher can limit the findings to. Furthermore, Scopus has functions like time span, source type, and type of documents. In each function, Scopus allows the researcher to limit and refine data in a reproducible way. For the current study, the reproducibility of data can be achieved by inserting the chosen keywords in advanced search in Scopus followed by refining results using the functions explained in details in subsequent paragraphs. At any time, the number of publications retrieved by any other researchers will be almost the same as presented in result section with very slight change that might be due to continuous updating of Scopus system. Regarding the total number of citations for retrieved articles, it is changeable with time because citation is a dynamic process. For this reason, all information pertaining to total number of citations and *h*-index are valid at the time specified in the study.

The first step and the most crucial one in any bibliometric analysis is the identification of keywords that will yield the highest number of retrieved articles with minimum false negative or false positive results. The selected keywords to be used in this study were those related to poverty which include: poverty or “out-of-pocket payments” or “catastrophic payments”. These selected keywords were entered in Scopus search engine in title search. The use of these keywords in title search aimed at increasing accuracy. However, these keywords are not considered enough to filter false negative results. Therefore, we added another condition to the search query. The keyword poverty was used again as a conditional term for the title search mentioned above. Therefore, Scopus will search for keywords like poverty or “out-of-pocket payments” or “catastrophic payments” in article title and will retrieve only articles in which the word “poverty” was also mentioned in abstract or keyword list. The ultimate search query looked like this: TITLE (poverty OR “out-of-pocket payments” OR “catastrophic payments”) AND TITLE-ABS-KEY (poverty). The keyword “food insecurity” could fit within the search query for this study. However, when “food insecurity” keyword was used, an extra 200 articles were retrieved. However, many of these articles were not poverty related and those that were poverty related were already retrieved. Therefore, the highest accuracy scenario was the one presented above that would achieve minimum acceptable error. Furthermore, poverty and food insecurity are not the same (Wight et al. 2014).

The second step in bibliometric analysis is to limit the time span of the study and to refine retrieved data. The time span for this study was set from year 2005 to year 2015. Research on poverty has been carried out for decades and we expect that the volume of literature on poverty will be huge. Therefore, specifying the time period from 2005 to 2015 will make the amount of data retrieved reasonable to handle. Furthermore, in the past decade or so, many wars, political unrest, internal conflicts were witnessed in most parts of the world especially Middle East and Africa and these wars created massive numbers of refugees and internally displaced people living in camps under poor conditions that can lead to various types of diseases. Therefore, we consider the specified period for the study to be justifiable and relevant.

The third step was to refine data by excluding books, book chapters and errata (correction) documents which can be accomplished easily using the refine and limit functionalities in Scopus. The purpose of this step was to restrict the analysis to literature in peer reviewed journals. Journal articles are considered original and novel work and that is why we focused on journal articles rather than books or book chapters. Furthermore, in any topic, the percentage of documents published as chapters or books is very small compared to journal articles. The validity of the search strategy was confirmed by manual review of a sample of 300 retrieved articles selected across the time span of the study.

The fourth step in the methodology of this study was to limit retrieved data to articles under subject area “Medicine”. Scopus classifies retrieved articles into different subject areas, one of which is “Medicine”. For the purpose of this study, only articles categorized under subject area ‘Medicine” were analyzed. Therefore, articles under “Medicine” subject area in which poverty was the main theme were retrieved and analyzed.

The fifth step in bibliometric analysis was to carry out analysis to present the required bibliometric indicators which include the followings: (1) types of published documents, (2) languages, (3) annual growth of publications, (4) citation analysis, (5) active countries, institutions, journals and authors, (6) International collaboration, (7) most frequent terms, and (8) highly cited articles. Some of these parameters were presented as tables, and some were presented as figures while others were presented as visualization map using VOSviewer technique (van Eck and Waltman 2010). For each of these parameters, further explanation will be presented in result section.

In this study, international collaboration was defined as an article being published by at least two authors with two different country affiliation. Of course, Scopus database has the country affiliation for each author in every published article and allows country analysis through tables or maps easily once that data are exported to Microsoft Excel software. In this regard, Scopus allows us to identify publications with multiple country affiliations and publications with single country affiliation. Single country publications (SCP) are articles in which all authors have the same country affiliation and such publications represent intra-country collaboration. Multiple country publications (MCP) are articles in which authors have different country affiliation and such publications represent inter-country collaboration.

For citation analysis, total citations for retrieved articles, average number of citations per article, percentage of highly cited articles, and Hirsch (*h*) index were presented (Hirsch 2005). These indicators are considered an indirect assessment of quality where high *h*-index and number of citations might be considered as an indicator of high quality. For assessing impact of publications in different journals, the impact journal (IF) of the publishing journal was used. The IF was retrieved from the latest Journal Citation Report published by Thompson Reuters. Highly cited articles were obtained from Scopus by sorting retrieved data based on number of citations then exporting whatever number of articles to Microsoft Excel for tabulation and then to Endnote for appropriate referencing. The standard competition ranking (SCR) was used to rank top ten active countries, institutions, and authors. Whenever necessary, data pertaining to SCP and MCP were also presented.

**Fig. 1:**
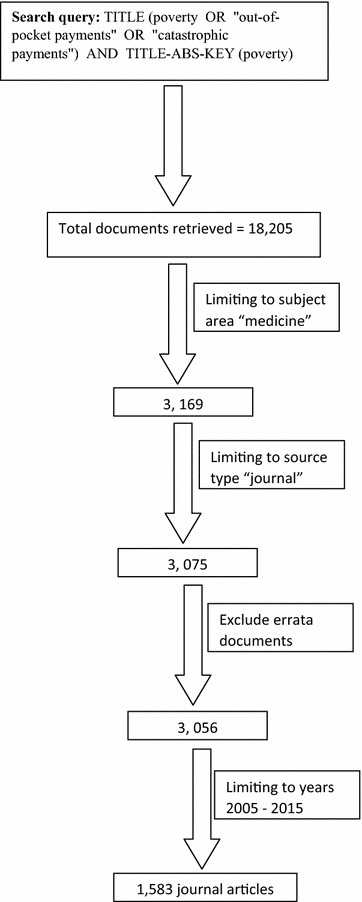
Scheme showing search strategy to obtain publications on medicine-related publications (2005–2015). Search strategy was based on keywords related to poverty and the results were refined based on subject “medicine”, source type “journal articles, time span “2005–2015” and excluding errata documents

The sixth and last step in bibliometric analysis is seeking ethical approval to carry out the study. In the case of bibliometrics, where no human subjects or data were involved, the institutional ethical committee asked for no ethical approval for such a study. Figure 1 shows a scheme for data retrieval for this study with numbers showing how many documents were retrieved in each step.

## Results

### General data

A total of 1583 journal articles were retrieved (Fig. 1). Two thirds (n = 1075; 67.91%) were research articles while the remaining were review articles, letters, notes, editorials, short surveys and articles in press. Table 1 shows the various types of retrieved documents. The total number of different languages used in publishing retrieved articles was 15. The primary language was English (1426; 90.08%). Other languages like French, Italian, German, Spanish, Portuguese, Polish, Japanese, Chinese and Turkish were also encountered. Table 2 shows the list of encountered languages in retrieved articles. Retrieved articles received a total of 17,131 citations, an average number of 10.82 citations per article. The *h*-index of retrieved articles was 56. A total of 1114 (70.37%) articles were cited at least once while the remaining 469 (29.63%) were not cited at all.

**Table 1:** Types of retrieved documents on poverty (2005–2015)

Type of document	Frequency	%
Article	1075	67.91
Review	154	9.73
Editorial	147	9.29
Note	87	5.50
Letter	49	3.10
Short survey	25	1.58
Conference paper	24	1.52
Article in press	22	1.39

**Table 2:** languages of retrieved articles on poverty (2005–2015)

Language	Total number of documents	(%)^a^
English	1426	90.08
Spanish	51	3.22
Portuguese	32	2.02
German	30	2.0
Chinese	27	1.70
French	23	1.45
Italian	9	0.57
(Norwegian, Czech, Danish, Swedish, Turkish, Croatian, Japanese, Korean, Slovenian)	16	1.07

^a^Total percentages might exceed 100% because some articles are written in 2 languages in the abstract and title and therefore some overlap might exist

### Publications with time

During the study period, the annual number of published articles on poverty increased slightly and in a fluctuating manner. The highest number of published articles was in 2007 with a total of 237 articles. Table 3 shows the number of retrieved articles per year along with their citation analysis. Table 3 shows that articles published in 2005 had the highest average number of citations per article while those published in 2015 had the least number of citations per article.

**Table 3:** Annual number of published articles and citations analysis (2005–2015)

Year	Total number of articles	%	TC	C/A	Number of articles with citations	%	Number of articles with no citations	%
2015	205	12.95	347	1.69	96	46.83	109	53.17
2014	179	11.31	565	3.16	109	60.89	70	39.11
2013	145	9.16	860	5.93	105	72.41	40	27.59
2012	124	7.83	890	7.18	93	75.00	31	25.00
2011	116	7.33	1478	12.74	89	76.72	27	23.28
2010	144	9.10	1999	13.88	113	78.47	31	21.53
2009	127	8.02	1936	15.24	97	76.38	30	23.62
2008	131	8.28	1956	14.93	110	83.97	21	16.03
2007	238	15.03	2791	11.73	161	67.65	77	32.35
2006	105	6.63	2467	23.50	81	77.14	24	22.86
2005	69	4.36	1822	26.41	58	84.06	11	15.94

*TC* total citations, *C/A* number of citations per article calculated by dividing the total number of citations retrieved for each year by the total number of publications in that year

### Most frequently encountered terms

**Fig. 2:**
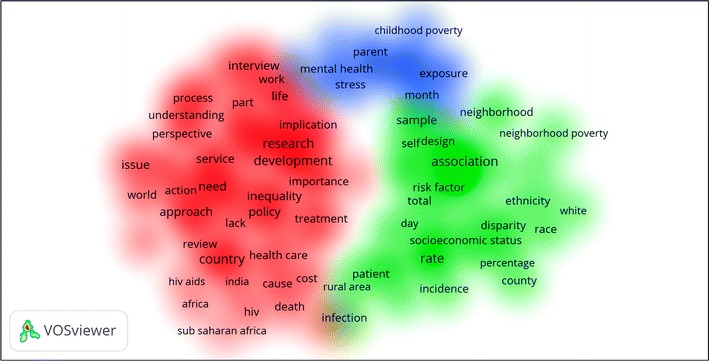
Density visualization map of most frequently related terms in retrieved articles

Retrieved articles were analyzed for most commonly encountered terms in title and abstract of retrieved articles. The frequent terms were visualized using VOSviewer. Figure 2 shows a visualization map of most frequently related terms. The map consists of three clusters of terms presented in different colors. The green cluster included terms such as ethnicity, socioeconomic status, neighborhood poverty, rural area and infection. The red cluster included terms such as Africa, India, HIV, cost, policy and infection. The blue cluster included terms such as childhood poverty and mental health.

### Countries

A total of 94 countries participated in publishing retrieved articles. Table 4 shows top 10 active countries in publishing medicine-related poverty documents. The top 10 active countries participated in publishing a total of 1187 (74.98%). The United States of America (USA) (589; 37.21%) had the greatest share of publications followed by the United Kingdom (UK) (174; 10.99%), and Canada (103; 6.51%). The USA was also the leading country in the annual number of publications from 2005 to 2015. More than half (54.5%) of worldwide articles on medicine-related poverty publications were produced by the USA, the UK and Canada. Countries like South Africa, Australia, Brazil, India, and China were also listed within the top 10 active countries.

**Table 4:** Top 10 active countries in number of publications (2005–2015)

SCR^a^	Country	Number of articles	%	NCC	MCP	%	SCP	%
1st	United States	589	37.21	62	127	21.56	462	78.44
2nd	United Kingdom	174	10.99	50	94	54.02	80	45.98
3rd	Canada	103	6.51	32	39	37.86	64	62.14
4th	South Africa	76	4.80	27	37	48.68	39	51.32
5th	Australia	59	3.73	31	28	47.46	31	52.54
6th	India	44	2.78	36	22	50.00	22	50.00
7th	Brazil	43	2.72	9	15	34.88	28	65.12
8th	China	39	2.46	19	18	46.15	21	53.85
9th	Germany	31	1.96	16	15	48.39	16	51.61
10th	Switzerland	29	1.83	33	26	89.66	3	10.34

*NCC* number of collaborating countries, *SCP* single country publication (intra-country collaboration), *MCP* multiple country publications (inter-country publications)

^a^ *SCR* Standard competition ranking. Equal countries were given the same ranking number, and then a gap is left in the ranking numbers

International (inter-country) collaboration was also shown in Table 4. Although the USA had the highest number of collaborating countries (n = 62), articles from USA had the least percentage of inter-country collaboration calculated as percentage of multiple country publication (MCP). For the USA, approximately 79% of articles were published by domestic authors presented as percentage of single country publication (SCP). On the other hand, approximately 90% of articles produced by researchers from Switzerland, for example, had co-authors from different countries. For the top 10 active countries a total of 421 (35.47%) articles were MCP suggestive of international collaboration while 64.53% of published articles by top 10 active countries were SCP.

### Authors and institutions

Top active authors who published most were from different countries (Table 5). No major dominance of any particular author was seen in this field and no single author had major contribution over other researchers. Analysis of top 10 active authors also showed that three authors were from the USA, three from Australia, two from Canada, one from South Africa and one from the UK. Similarly, there were no major dominance of any particular institution for research in poverty over other institutions, although *London School of Hygiene & Tropical Medicine* in the UK ranked first in number of publications, number of citations and *h*-index (Table 6). The difference between the top 10 active intuitions in the number of publications was not large.

**Table 5:** Top 10 prolific authors publishing on poverty-related health issues (2005–2015)

SCR^a^	Author	Frequency	%	Country
1st	Hotez, P.J.	16	1.01	USA
2nd	Callander, E.J.	11	0.69	Australia
2nd	Schofield, D.J.	11	0.69	Australia
4th	Séguin, L.	10	0.63	Canada
5th	Lund, C.	9	0.57	South Africa
5th	Schootman, M.	9	0.57	USA
5th	McClellan, W.M.	9	0.57	USA
8th	Shrestha, R.N.	8	0.51	Australia
9th	Emerson, E.	7	0.44	UK
9th	Gauvin, L.	7	0.44	Canada

*USA* United States of America, *UK* United Kingdom

^a^ *SCR* Standard competition ranking. Equal countries were given the same ranking number, and then a gap is left in the ranking numbers

**Table 6:** Top 10 productive institutions on poverty in health-related journals (2005–2015)

SCR^a^	Institution	Frequency	%	Country affiliation	TC	*h* index
1st	*London School of Hygiene & Tropical Medicine*	34	2.15	UK	848	18
2nd	*The University of North Carolina at Chapel Hill*	31	1.96	USA	577	12
3rd	*Universite de Montreal*	24	1.52	Canada	149	7
3rd	*Harvard School of Public Health*	24	1.52	USA	701	13
5th	*University of Cape Town*	23	1.45	South Africa	327	8
6th	*University of California, San Francisco*	22	1.39	USA	299	10
6th	*Organisation Mondiale de la Sante*	22	1.39	WHO	929	11
8th	*University of Toronto*	21	1.33	Canada	235	8
9th	*University of Illinois at Chicago*	19	1.20	USA	774	11
10th	*Johns Hopkins Bloomberg School of Public Health*	18	1.14	USA	485	11
10th	*University of KwaZulu*-*Natal*	18	1.14	South Africa	217	8
10th	*Centers for Disease Control and Prevention*	18	1.14	CDC/USA	597	9
10th	*The University of Sydney*	18	1.14	Australia	55	4

*TC* total citations, *h*-*index* Hirsch index, *USA* United States of America, *UK* United Kingdom, *WHO* World Health Organization, *CDC* Centers for Disease Control and Prevention

^a^ *SCR* standard competition ranking. Equal countries were given the same ranking number, and then a gap is left in the ranking numbers

### Highly cited articles

The top 20 highly cited articles on medicine-related poverty publications were presented in Table 7. Of the top 20 list, 14 were research articles and four were review articles, one was editorial and one was a conference paper. The article which received the highest number of citations was “Neighborhood racial composition, neighborhood poverty, and the spatial accessibility of supermarkets in metropolitan Detroit” (Zenk et al. 2005) published in *American Journal of Public Health*. The article received a total of 439 citations up to the time of data analysis (September 15, 2016). The topics covered in top 20 cited articles were infectious diseases, mental health, nutrition, and development as related to poverty.

**Table 7:** Top 20 cited articles on poverty-related issues (2005–2015)

References	Title	Number of citations	References	Title	Number of citations
Zenk et al. (2005)	Neighborhood racial composition, neighborhood poverty, and the spatial accessibility of supermarkets in metropolitan Detroit	439	Lund et al. (2011)	Poverty and mental disorders: breaking the cycle in low-income and middle-income countries	122
Alvar et al. (2006)	Leishmaniasis and poverty	255	Katabira and Oelrichs (2007)	Is poverty or wealth driving HIV transmission?	121
King (2010)	Parasites and poverty: the case of schistosomiasis	226	Emerson (2007)	Poverty and people with intellectual disabilities	119
Farah et al. (2006)	Childhood poverty: Specific associations with neurocognitive development	226	Yoshikawa et al. (2012)	The effects of poverty on the mental, emotional, and behavioral health of children and youth	112
van Doorslaer et al. (2006)	Effect of payments for health care on poverty estimates in 11 countries in Asia: an analysis of household survey data	220	Galea et al. (2007)	Urban neighborhood poverty and the incidence of depression in a population-based cohort study	112
Miech et al. (2006)	Trends in the association of poverty with overweight among US adolescents, 1971–2004	168	Tanumihardjo et al. (2007)	Poverty, obesity, and malnutrition: an international perspective recognizing the paradox	105
Duncan et al. (2010)	Early-childhood poverty and adult attainment, behavior, and health	146	Subramanian et al. (2005)	Racial disparities in context: a multilevel analysis of neighborhood variations in poverty and excess mortality among black populations in Massachusetts	101
Hotez (2008)	Neglected infections of poverty in the United States of America	138	Blair et al. (2011)	Salivary cortisol mediates effects of poverty and parenting on executive functions in early childhood	98
Smith et al. (2006)	The infant development, environment, and lifestyle study: Effects of prenatal methamphetamine exposure, polydrug exposure, and poverty on intrauterine growth	138	Hotez and Wilkins (2009)	Toxocariasis: America’s most common neglected infection of poverty and a helminthiasis of global importance?	95
Nandy et al. (2005)	Poverty, child undernutrition and morbidity: new evidence from India	126	Choi and Holroyd (2007)	The influence of power, poverty and agency in the negotiation of condom use for female sex workers in mainland China	93

### Journals

The total number of different journal names which published a minimum of three articles on poverty was 117. A total of 316 (19.96%) articles were published in the top 20 active journals (Table 8) which suggests that there was no major dominance of any particular journal over others in publishing articles on poverty-related health issues. In fact, most of the journals in the top 20 list were either in general medicine or in public health field. The journal that has the largest share of publications was *Lancet* (n = 32) while the *American Journal of Public Health* received the greatest number of citations and *Tropical Medicine and International Health* journal had the highest average number of citations per article. Table 8 also shows the IF for the top 20 active journals. The highest IF value in 2015 was that of *Lancet* which had an IF value of 44. All journals in the top 20 list are indexed in ISI Thompson Reuters and some of them had high IF suggestive of the great medical importance of poverty on individual and population health.

**Table 8:** Top 20 health-related journals publishing on poverty-related health issues (2005–2015)

SCR^a^	Journal	Frequency	%	TC	C/A	IF
1st	*Lancet*	32	2.02	620	19.38	44.002
2nd	*American Journal of Public Health*	26	1.64	864	33.23	2.24
3rd	*Plos One*	25	1.58	207	8.28	3.54
4th	*Health and Place*	20	1.26	254	12.70	1.74
5th	*Social Science and Medicine*	19	1.20	530	27.89	2.814
6th	*BMC Public Health*	18	1.14	216	12.00	2.209
7th	*Plos Neglected Tropical Diseases*	18	1.14	564	31.33	4.446
7th	*Journal of Epidemiology and Community Health*	16	1.01	220	13.75	3.31
9th	*BMJ Clinical Research Ed* ^b^	15	0.95	17	1.13	17.4
10th	*Ciencia E Saude Coletiva*	14	0.88	71	5.07	0.50
11th	*Bulletin of The World Health Organization*	13	0.821	435	33.46	5.089
12th	*International Journal for Equity in Health*	12	0.758	70	5.83	2.378
13th	*Tropical Medicine and International Health*	11	0.695	386	35.09	2.519
14th	*Journal of Urban Health*	10	0.632	154	15.40	2.046
14th	*Paediatrics and Child Health*	10	0.632	46	4.60	1.477
14th	*Pediatrics*	10	0.632	320	32.00	5.473
14th	*Preventing Chronic Disease*	10	0.632	51	5.10	2.170
14th	*Salud Publica De Mexico*	10	0.632	35	3.50	1.107
19th	*International Journal of Health Services*	9	0.569	42	4.67	0.782
19th	*Journal of Health Care for The Poor and Underserved*	9	0.569	52	5.78	0.924
19th	*Plos Medicine*	9	0.569	225	25.00	14.429

*TC* total citations, *C/A* average number of citations per article calculated by dividing the total citation by number of articles for each journal, *IF* impact factor

^a^ *SCR* standard competition ranking. Equal countries were given the same ranking number, and then a gap is left in the ranking numbers

^b^Discontinued in 1988. Currently it is known as *BMJ* (*British Medical Journal*)

## Discussion

In this study, a bibliometric overview of medicine-related poverty publications was presented. The use of Scopus search engine in this study was justifiable given the advantages it has over other databases (Falagas et al. 2008). Literature search identified two studies on bibliometrics as related to poverty. However, these two studies were directed to either assessment of collaboration between European countries and African countries on research pertaining to neglected tropical and infectious diseases or toward implementation of research on diseases of poverty (Gonzalez-Block et al. 2011; Breugelmans et al. 2015). Neither of these studies assessed the worldwide publications on poverty and its relation to health. It should be emphasized that there are thousands of publications on poverty in non-medical subject area such as politics, economy, social studies and religion that were not considered in this study. In our study, we focused on poverty literature within the subject “Medicine”.

The number of publications slightly increased over the study period. It is evident that most governmental and non-governmental funding goes to research domains in infectious diseases, nutritional disorders and neuro-developmental growth but not to poverty per se. The peak of research productivity seen in 2007 is difficult to explain and we are unable to determine whether this peak is due to a surge in domestic violence and wars with the spread of thousands of refugees and poverty or it is just an unexplained coincidence. Despite all this, most publications about poverty were published in highly prestigious and influential journals such as *Lancet*. Furthermore, the importance of “poverty” as a research topic in social sciences, psychology and community health was emphasized by the introduction of specialized journals such as *Journal of Poverty, Journal of Poverty and Social Justice,* and *Journal of Children and Poverty.* In this study, no major dominance of any particular journal regarding number of publications was seen suggesting that poverty is a wide concept affecting various health issues. For example, many journals in the field of parasitology and infectious diseases published articles on poverty (Alvarado-Esquivel et al. 2013). Similarly, many journals in the field of nutrition, psychology and public health published similar articles on poverty and its relation to health (Bhattacharya et al. 2004). Finally, the presence of non-English articles is another indicator of worldwide growing interest in the medical and social aspects of poverty.

Analysis of publications showed that countries in Latin America, Africa, and Asia had contributed to this field (Szwarcwald et al. 2002; Das et al. 2007). This is not surprising given that poverty is common in countries like India, Brazil, and Africa in general. International collaboration among the top 10 active countries was modest with approximately approximately one third of articles had multiple country affiliation. International collaboration in research is highly needed and should be encouraged since most low and middle income countries cannot fund such research and might not have the advanced technical and medical tools used to investigate poverty-related diseases or health issues. Furthermore, international collaboration increases the quality of publications and chances of articles to be cited and published in high impact journals (Huamani et al. 2015).

Analysis of top cited articles revealed that most retrieved articles addressed issues of neglected diseases, particularly parasitic diseases. Other topics found in top cited articles were those pertaining to nutrition, child psychology and mental development. The number of people being affected with infectious diseases in low and middle income countries is high and many countries have implemented health policies to control such common diseases by reducing poverty rates (Calisher 2007; Huntington 2012). It was not surprising that such articles were published in highly prestigious general journals simply because it is estimated that one billion people worldwide are living below the poverty line. As expected, the *World Health Organization* and its affiliated Journal (*Bulletin of the World Health Organization*) had a good share of publications in this field.

Density visualization map showed that a group of articles had linked poverty with development, neurocognitive, intelligence, behavioral, and mental health. A study had shown that higher socioeconomic status was associated with better performance on neurocognitive tests (Farah et al. 2006). Another study had linked poverty with childhood brain development and academic achievement. The authors of that study argued that families with low income should be targeted to avoid heavy cost of poor academic achievement (Hair et al. 2015). The association between family poverty and mental health and behavior of children have been investigated and authors argued that mental health programs need to be scaled up (Lund et al. 2011). Poverty have been associated with drinking alcohol, violence, smoking, stress and depression (Grant et al. 2005; Lovisi et al. 2005; Haustein 2006; Wheeler et al. 2006; Perese 2007; Mossakowski 2008; Mulia et al. 2008; Tracy et al. 2008; Kinyanda et al. 2011; Nikulina et al. 2011).

Another group of articles on poverty have linked poverty with infectious diseases, particularly HIV and parasitic diseases. Of these particular infectious diseases, leishmaniasis, schistosomiasis, malari, toxocariasis and tuberculosis were strongly linked to poverty (Grant et al. 2005; Lovisi et al. 2005; Haustein 2006; Wheeler et al. 2006; Perese 2007; Mossakowski 2008; Mulia et al. 2008; Tracy et al. 2008; Kinyanda et al. 2011; Nikulina et al. 2011). The association between poverty in one hand and drug abuse and HIV on the other hand have been extensively investigated. A study claimed that chances of poor people being exposed to HIV are not necessarily greater than wealthier individuals or households and that HIV hit across all socioeconomic strata (Katabira and Oelrichs 2007). The association between poverty and HIV/AIDS is best exemplified by the situation in Africa (Kalichman et al. 2005; Tladi 2006; Mbirimtengerenji 2007; Nattrass 2009; Thurlow et al. 2009; Fox 2010; Shisana et al. 2010; Tsai et al. 2013). Even some of the serious global viral diseases like Ebola and Dengue have been linked to poverty (Khun and Manderson 2008; Fallah et al. 2015). Association between poverty and infectious diseases is not present only in Africa but even in Europe, India, China, Latin America and the USA (Jackson et al. 2006; Riley et al. 2007; Silveira et al. 2008; Gryseels et al. 2009; Hotez 2010; Hotez and Gurwith 2011; Cooper et al. 2012; Dowd et al. 2012; Hotez et al. 2012; Karan et al. 2012; Oxlade and Murray 2012; Bhutta et al. 2014; Hotez et al. 2014; Yang et al. 2015).

A third group of articles on poverty focused on the association of poverty with nutrition and physical health. Actually most of the points suggested by the Millennium Development Goals issued by the United Nations were directly or indirectly related to nutrition and, in fact, the first goal in the plan was to eradicate hunger (Tanumihardjo et al. 2007). Studies have shown that poverty or food insecurity is associated with lower quality diets which might affect the biological health of people and their life span (Champagne et al. 2007; Crimmins et al. 2009). Vulnerable groups of people like pregnant women are highly affected by poverty and food insecurity (Braveman et al. 2010). Poverty have been linked to increased prevalence of kidney diseases, diabetes, diabetic foot amputation, hypertension and cardiovascular diseases, and osteoporotic fractures (Wachtel 2005; Seedat 2007; Navarro et al. 2009; McClellan et al. 2010; Hsu et al. 2012; Booth et al. 2013; Gaskin et al. 2014). Obesity have also been associated with poverty and such association might be due to lack of physical activity or consumption of larger amounts of carbohydrates (Miech et al. 2006; Prentice and Webb 2006; Drewnowski et al. 2009; Ziol-Guest et al. 2009; Usfar et al. 2010).

A fourth dimension in retrieved articles was health policies to face poverty as global or national public health burden (McGarry and Schoeni 2005; Mahmud Khan et al. 2006; Meessen et al. 2006; Woolf et al. 2006; Ferguson et al. 2007; Limwattananon et al. 2007; Khun and Manderson 2008; Kruk et al. 2008; Zimmer 2008; Garg and Karan 2009; Schneider et al. 2009; Falkingham et al. 2010; Leatherman and Dunford 2010; Shahrawat and Rao 2012; Bhutta et al. 2014). Such articles focused on raising calls for implementation of economic plans for better distribution of national wealth to help poor people to get access to medicines and healthcare. The above mentioned main dimensions of retrieved articles were visualized in the density visualization map presented in Fig. 2.

Our study has few limitations related to nature of bibliometric studies (Sweileh et al. 2013, 2014; Zyoud et al. 2015a, b, c, d). False positive and false negative results are difficult to avoid regardless of how accurate the search stagey was. However, we believe that false positive or negative results were very marginal and could hardly affect the accuracy of the results of our study. Search strategy using title search increased the accuracy and minimized false positive results. One might argue against such strategy, but we thought it will be unfair to include all articles with the keyword “poverty” in title-abstract-keywords. Limiting search query to article title increased the accuracy of retrieved articles.

## Conclusions

To the best of our knowledge, this is the first worldwide bibliometric study on poverty publications in relation to medicine. The results of our study showed that poverty research has been slowly progressing without any major or significant dominant leadership for any institution or author or journal. However, the leadership of the USA in the number of publications was evident. There is a prominent focus on poverty research in relation to infectious diseases and child development as demonstrated by the title of top cited articles. Given the global burden of poverty, inter-country collaboration among the top active found in this study is not up to the global challenge of poverty.

## Abbreviations

*h*-index: the Hirsch index
IF: impact Factor
USA: Unite States of America
UK: United Kingdom
WHO: World Health Organization
